# Safety of a Novel ESAT6-CFP10 skin test compared with tuberculin skin test in a double-blind, randomized, controlled study

**DOI:** 10.1186/s12879-022-07765-w

**Published:** 2022-10-11

**Authors:** Yang Yang, Zhixiong Fang, Wei Huang, Haiming Zhang, Si Luo, Sha Lin, Shaojie Li, Shuihua Lu

**Affiliations:** 1 Shanghai Public Health Clinical Center Affiliated to Fudan University, 201508 Shanghai, China; 2 Department of Infectious Disease and Public Health, Central Hospital of Xiangtan, Xiangtan, Hunan province China; 3 Xiangtan Center for Disease Control and Prevention, Xiangtan, Hunan province China; 4 Department of General Surgery, Xiangtan First People’s Hospital, Xiangtan, Hunan province China; 5 Department of Pulmonary Medicine, The Second Affiliated Hospital, School of Medicine, National Clinical Research Center for Infectious Disease, Shenzhen Third People’s Hospital, Southern University of Science and Technology, 518112 Shenzhen, Guangdong Province China

**Keywords:** Tuberculosis, ESAT6-CFP10, Safety, Tuberculin skin test

## Abstract

**Background:**

ESAT6-CFP10 (EC) skin test has been reported accurate and safe in identifying tuberculosis infection. We aimed to demonstrate the safety of EC skin test compared with tuberculin skin test (TST) in university freshmen.

**Methods:**

We conducted a double-blind, randomized, controlled clinical study in a university freshmen population with 16,680 participates in China, and finally 14,579 completed the study. About a half received an EC skin test and the others received TST. Adverse reactions were evaluated.

**Results:**

Out of the 14,579 participants, 48.2% (7029/14,579) were males. The average age was 18.1 ± 0.8 years and the average BMI was 20.9 ± 3.1 kg/m^2^. 50.4% (7351/14,579) participants received EC skin test and 49.6% (7228/14,579) received TST. The EC group had significantly less adverse reactions compared with the TST group (21.3%, 1565/7351 vs. 34.6%, 2499/7228, *P = 0.000*). The most common adverse reactions for EC were bleeding (5.63%, 414), dermatodyschroia (4.27%, 314), induration (3.90%, 287), swelling (2.49%, 183), pain (1.59%, 117) and pruritus (1.48%, 109). Bleeding, dermatodyschroia, swelling and erythema were significantly less in EC group (*P < 0.05*), while others were similar to those of TST.

**Conclusion:**

the EC skin test was safe in our cohort. And its incidence of total adverse drug reactions (ADRs) is less than that of TST. Most adverse reactions were mild or moderate, lasting less than 48 h and self-limiting. Considering the satisfactory diagnostic accuracy in identifying tuberculosis infection, the cost and safety, the EC skin test might be a potential candidate for replacing TST in high burden countries or those with routine BCG vaccination. Clinical Trials Registration. ChiCTR2000038622, Safety of the EC skin test to screen tuberculosis infection in two universities, compared with the tuberculin skin test: a double-blind, randomized, controlled trial. registered on 26/09/2020 at http://www.chictr.org.cn.

## Background

Tuberculosis is one of the top 10 causes of death worldwide and the leading cause of death from a single infectious agent (ranking above HIV/AIDS) [1]. Approximately 10 million people fell in with TB and it was responsible for over 1 million death each year globally in recent ten years. About a quarter of the world’s population is infected with *Mycobacterium tuberculosis*, and it is estimated their lifetime risk of developing tuberculosis is 5-10% [2]. In order to effectively control the epidemic of TB, scanning out people with tuberculosis infection is important. Two currently available diagnostic tool for tuberculosis infection are interferon-gamma release assays (IGRAs), including T-SPOT.TB test and QuantiFERON-TB Gold In-Tube, and the tuberculin skin test (TST) [3, 4]. Compares with TST, IGRAs is more specific because it is less likely to be affected by Calmette Guerin (BCG) vaccination or exposure to nontuberculous mycobacteria[5–7]. Since over 95% of tuberculosis cases are in developing countries, IGRAs is not an affordable choice. Thus, TST is widely used in high-burden countries for its high sensitivity and low cost. While its poor specificity caused by prior BCG vaccination and exposure to nontuberculous mycobacteria results many false positive cases. ESAT6-CFP10 (EC) skin test is based on tuberculosis specific antigens and comparable to the T-SPOT.TB test on diagnostic accuracy [8]. In addition, considering the low cost of EC skin test, it is a promising tool to replace TST in high burden countries or those with routine BCG vaccination. It has been reported EC skin test had a good safety profile [8, 9]. In our study, we further explored the safety of EC skin test compared with that of TST.

## Methods

### Study Design and participants

This was a double-blind, randomized, controlled clinical study conducted in college freshmen in Xiangtan University and Hunan University of Science and Technology in Xiangtan City, Hunan Province, China. The inclusion criteria were: 1) willing to receive EC or TST testing and provide information on past medical history. The exclusion criteria were: (1) had been vaccinated with any live vaccine within the preceding 6 months. (2) had received TST in the previous 12 months (to eliminate the potential risk for boosting). (3) had received Immunosuppressive therapy treatment. (4) refusing to participate in this study. 4) could not provide written informed consent. (5) could not complete all surveys and testing items. (6) current skin conditions that could interfere with the measurement of induration after the EC test or TST. (7) were currently participating in other drug clinical trials. (8) other disorders that investigators deemed unsuitable.

## Randomization and masking

Participants were enrolled and randomly assigned into two groups at 1:1 (EC group and TST group). The randomization schedule was generated centrally by an independent statistician not involved in the study, by random permutation programmed in SPSS Statistics version 25. Investigators and participants were masked to skin test allocations. To mask which test was being injected in forearm, physicians were provided with test kits that contained two kinds of identical vials, one containing EC and one containing TST, and instructions about in which participant each test should be administered. The vials and the cardboard box of each kit were all labelled with the same randomization number.

## Procedures

The EC antigen is a recombinant reagent of the ESAT-6 and CFP-10 tests, developed by Zhifei Longcom Biologic Pharmacy Company, China. The TST (TB-PPD) was manufactured by Beijing Gaoke Life and Technology, China, and administrated by trained nurses, following the national standard guideline [10]. Both the EC and TST agent were clear, colorless solutions. Participants received the EC skin test (1.0 µg/0.1 mL) [8] or TST on the volar surface of one forearm. Injection site ADRs such as bleeding, swelling, pain, pruritus, erythema, blistering and ulceration, systemic ADRs (all non-injection-site reactions) such as fatigue, headache, nausea, vomiting and palpitation were assessed by investigators at 30 min, 24 h, 48 h, 72 h after skin test, respectively.

## Follow-up and data collection

Participants attended follow-up visits on 24 h, 48 h, 72 h and day 28 after skin test, respectively for evaluation of skin test ADRs. The evaluation of the results was done by trained doctors, and they were all blinded about the groups of intervention. General information such as age, gender, height and weight, as well as medical history were obtained and recorded by investigators after the assignment of written informed consent.

### Statistical analysis

Numerical data are presented as number (%) of participants, mean (SD). Categorical data are presented as number (%) of participants. Differences in number of participants, gender, and adverse reactions between EC group and TST group were evaluated with χ2 test, and Continuity Correction or Fisher’s Exact test would be used when necessary. Differences in age and body-mass index (BMI) between EC group and TST group were evaluated with t test. Statistical analyses were done with SPSS Statistics version 25. This study is registered with http://www.chictr.org.cn (ChiCTR2000038622).

## Results

**Fig. 1:**
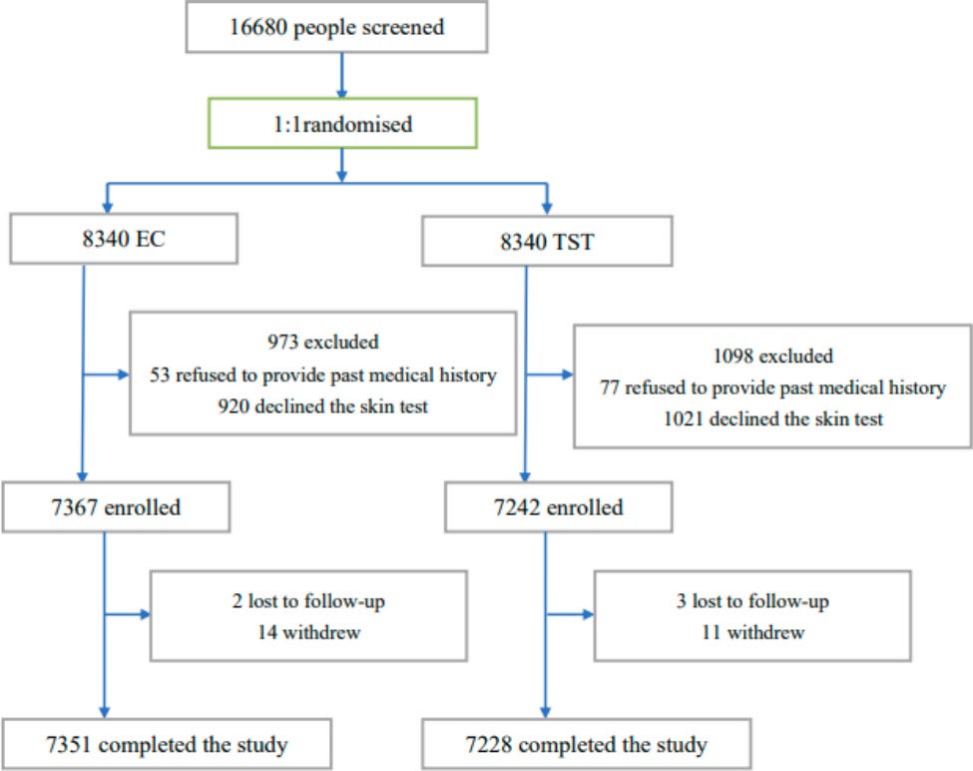
Trial Flow. EC = EAST6-CFP10; TST = tuberculin skin test.

From October 15, 2020, to October 30, 2020, 16,680 healthy freshmen were screened and all participants or their legal guardians signed written informed consent. They were randomly assigned to the EC group or TST group. 973 and 1098 participants in the EC and TST group were excluded and 7363 and 7242 were enrolled, respectively. Finally, 14,579 participants completed the study. 7351/14,579(50.4%)and 7228/14,579 (49.6%) were analyzed in the EC and TST group, respectively (Fig. 1). In all the 14,579 participants, males accounted for 7029/14,579 (48.2%). The average age was 18.1 ± 0.8 years. The average BMI was 20.9 ± 3.1 kg/m^2^. The EC group had significantly less adverse reactions compared with the TST group (21.3%, 1565/7351 vs. 34.6%, 2499/7228, *P = 0.000*). Proportions of ADRs of EC and TST are listed in Table 1. In the EC group, common ADRs (≥ 1.0% and < 10.0%) were bleeding, dermatodyschroia, induration, swelling, pain and pruritus. Uncommon ADRs (≥ 0.1% and < 1.0%) were erythema, blistering, fatigue, headache and injection site rash. Rare ADRs (≥ 0.01% and < 0.1%) were nausea, dyspnea, vomiting, body ache, palpitation and ulceration. In the TST group, common ADRs (≥ 1.0% and < 10.0%) were induration, bleeding, dermatodyschroia, swelling, pain, pruritus and erythema. Uncommon ADRs (≥ 0.1% and < 1.0%) were blistering, injection site rash, fatigue, headache and nausea. Rare ADRs (≥ 0.01% and < 0.1%) were dyspnea, vomiting, palpitation, ulceration and body ache. Compared with the ADRs of TST group, bleeding, dermatodyschroia, induration, swelling and erythema were significantly less in the EC group *(P < 0.05*, see Table 1*)*. All of the ADRs arose during the first 24 h after injection and were mild to moderate and lasted less than 48 h and were and self-limiting. In addition, no related serious ADRs were found during follow-up.

**Table 1:** characteristics and adverse reactions of participants

	EC	TST	*P*
number	7351 (50.4%)	7228 (49.6%)	
male	49.1% (3606/7351)	47.4% (3423/7228)	0.04
age	18.1 ± 0.8	18.1 ± 0.7	0.77
BMI	20.9 ± 3.1	20.9 ± 3.2	0.99
total ADR	1565 (21.3%)	2499 (34.6%)	0.000
bleeding	414 (5.63%)	467 (6.46%)	0.036
dermatodyschroia	314 (4.27%)	440 (6.09%)	0.000
induration	287 (3.90%)	933 (12.91%)	0.000
swelling	183 (2.49%)	258 (3.57%)	0.000
pain	117(1.59%)	115(1.59%)	0.998
pruritus	109 (1.48%)	112 (1.55%)	0.742
erythema	48(0.65%)	76(1.05%)	0.009
blistering	30 (0.41%)	27 (0.37%)	0.738
fatigue	18 (0.24%)	12 (0.17%)	0.294
headache	10 (0.14%)	12 (0.17%)	0.641
rash	9 (0.12%)	14 (0.19%)	0.278
nausea	6 (0.08%)	11 (0.15%)	0.212
dyspnea	6 (0.08%)	5 (0.07%)	0.784
vomiting	5 (0.07%)	5 (0.07%)	1.000^*^
body ache	4 (0.05%)	3 (0.04%)	1.000 ^#^
palpitation	3 (0.04%)	5 (0.07%)	0.504 ^#^
ulceration	2 (0.03%)	4 (0.06%)	0.449 ^#^

*: Continuity correction; #: Fisher exact test

## Discussion

Our study further explored the safety of EC skin test in a large size population, with 14,579 participants. Out of the 14,579 participants, the EC group had less adverse reactions compared with the TST group (21.3%, 1565/7351 vs. 34.6%, 2499/7228, *P = 0.000*). EC skin test has satisfactory diagnostic accuracy and high consistency (96.3%, 95% CI, 92.0-100.0) with the T-SPOT.TB test in identifying tuberculosis infection, which means an increased specificity over traditional TST. At present, the cost of EC skin test is about 10% of T-SPOT.TB test, 14.3% of QuantiFERON-TB Gold test, 2 times of TB-pure protein derivative (PPD) and 9 times of BCG-PPD. The efficiency, effectiveness and cost of EC skin test refers it the potential replacing the extensive usage of TST in countries with high burden or with routine BCG vaccination, which are also low-to-moderate income countries[8]. Phase I and II clinical trials of EC skin test have demonstrated it is safe in both healthy people and TB patients aged between 18 and 65, without any serious adverse reaction [8, 9, 11]. In the phase I clinical trial of EC skin test [9], 24 healthy volunteers aged 18 to 40 were enrolled to evaluate its safety, and the incidence of ADRs was 4.2% (1/24). The volunteer developed a mild local reaction of red spots scattered at the injection site 15 min after the skin test and disappeared on the next day. In the phase IIa clinical trial of EC skin test [11], 56 healthy adults and 88 TB patients aged 18 to 65 were enrolled and all received EC skin test. Besides, all healthy participants and 56 out of 88 TB patients received TB-PPD skin test at the same time while the rest 32 patients received placebo. In this IIa trial, the main ADRs for EC and TB-PPD skin test were local pruritus and pain. The incidence of the two ADRs in EC and TB-PPD were17.4% (25/144) and 22.3% (25/112), respectively, but without statistically difference. When it comes to the IIb clinical trial [8], 777 healthy participants, 96 TB patients and 95 nontuberculosis patients with other pulmonary disease whose age were between 18 and 65 were analyzed. The rate of ADRs of EC skin test and TST were 27.8% and 16.5% (*P = 0.000*), respectively. For both the EC skin test and the TST, pruritus and pain at the injection site were the most common ADRs, which was consistent to that in the phase IIa trial. In summary of all previous trials and our study, all the EC-related ADRs were mild to moderate and self-limiting, arising during the first 24 h after injection and lasting less than 48 h. No serious ADRs related to EC skin test was observed. Besides, in our study, which has a much larger sample size of participants, the rate of total ADRs in EC skin test group is statistically lower than that in the TST group. Instead of pruritus and pain at injection site in the previous studies, bleeding and dermatodyschroia were the most common ADRs of EC group while induration and bleeding were the most common ADRs of TST group in our study. Since other ADRs except for injection site pruritus and pain of EC in previous clinical trials were not known, we are not able do the comparison. The different sample size (the number of participants) and the selection of participants (healthy or patients, common adults or university freshmen) might be an explanation. The strength of our study included: (1) large number of participants. (2) easy procedure with good repeatability. (3) minimal loss to follow up with 5 participants lost to follow up and 25 withdrew out of 14,609 enrolled participants in total. The limitation in our study is the population was constrained to university freshmen. In future studies, wider population should be included.

## Conclusion

the EC skin test was safe in participants from our cohort. And its incidence of total ADRs is less than that of TST. Most adverse reactions were mild or moderate, lasting less than 48 h and self-limiting. Considering the satisfactory diagnostic accuracy in identifying tuberculosis infection, the cost and safety, the EC skin test might be a potential candidate for replacing TST in high burden countries or those with routine BCG vaccination.

## Data Availability

The datasets generated and/or analyzed during the current study are not publicly available due to Confidentiality Agreement, but are available from the corresponding author on reasonable request.
